# Metformin and renal injury protection

**DOI:** 10.12861/jrip.2013.29

**Published:** 2013-09-01

**Authors:** Mahmoud Rafieian-Kopaie

**Affiliations:** ^1^Medical Plants Research Center, Shahrekord University of Medical Sciences, Shahrekord, Iran

**Keywords:** Metformin, Diabetic nephropathy, Kidney

**Implication for health policy/practice/research/medical education:**

Metformin protects against tubular injury by modulation of oxidative stress on the tubules and restoring the biochemical alterations. While in diabetes, glycosuria causes tubular cell injury. Furthermore metformin is able to protect podocytes in diabetic nephropathy. These findings may suggest the clinical use of metformin in the prevention of diabetic nephropathy.

New indications for metformin have increased its popularity (1). Metformin is a biguanide drug which improves the sensitivity to insulin, increases the insulin-stimulated uptake and utilization of glucose, reduces basal hepatic glucose production, causes weight reduction and decreases hunger (1,2). Recent studies have recommended the use of this drug for kidney protection. These studies have suggested that metformin has antioxidant activities, too (3-5). The apoptosis, induced by oxidative stress, in endothelial cells was reduced and the vascular dysfunction was prevented following metformin therapy (2,5). It has been shown that gentamicin-induced renal tubular damage is reduced by metformin (5). To better understand the effect of metformin against gentamicin tubular toxicity, a study was conducted by us on male Wistar rats, in which we found metformin was able to prevent acute kidney injury induced by gentamicin. It indicates that metformin may have beneficial effects in patients who are under treatment with this drug (6). Recently, the ameliorative effect of metformin was demonstrated against ischemia–reperfusion induced injury in rats (7), which is in agreement with the results of our study. To test the effect of co-administration of metformin and garlic extract against gentamicin-renal toxicity in Wistar rats, we conducted a study on 70 male rats (8). The result of this study indicated that garlic or metformin and their co-administration has both protective and curative effects against gentamicin-induced nephrotoxicity. Therefore, metformin might be used together with garlic extract to increase the antioxidant efficacy and ameliorate gentamicin tubular toxicity (8-11). The enzyme AMP-activated kinase (AMPK), which regulates cellular and organ metabolism, has been shown to be associated with the pleiotropic actions of metformin (8-11). AMPK is a phylogenetically conserved threonine/serine protein kinase which is considered as fuel gauge monitoring systemic and cellular energy condition (5-9). It plays an important role in protecting cellular functions under energy-restricted circumstances (5-9). Various studies have suggested that AMPK activation by metformin is secondary to its effect on the mitochondria as the primary target of this agent (4-10). Some other studies have suggested mitochondrial activity for metformin (10,11). Indeed, there is evidence that, when metformin is used alone, the beneficial effect of metformin might be due to its mild inhibition of the mitochondrial respiratory chain (5-10). It is also suggested that metformin treatment may attenuate the increase in malondialdehyde and total reactive oxygen species generation and restore the decrease in both enzymatic and non-enzymatic antioxidants (5,10). Therefore, as we observed in the above addressed studies, metformin may impose its effects against kidney toxicity (5-8). However, the main question is, whether these experimental findings are applicable in clinical studies. We are unanimous for metformin to use it as a first-line glucose-lowering agent (1,2,11). It should be noted that due to increased risk of lactic acidosis, it could not be used in a proportion of patients with type 2 diabetes (1,2,11). Scientists suggest that it should be discontinued when estimated glomerular filtration rate is less than 30 mL/minute and be used with caution in estimated glomerular filtration rates of below 60 mL/minute (1,2,11,12). Lactic acidosis is a metabolic disorder with high mortality and in most of cases patients may need renal replacement (1,2,11,12). However, the risk of lactic acidosis might be decreased by avoiding metformin use in patients with hypovolemia, high risk of renal impairment, sepsis and reduced kidney capacity such as old age patients. Nevertheless, in these conditions, metformin might indeed act as an innocent bystander (1,2,11,12). Also in the recent studies on the relationship between metformin and cardiac insufficiency it was demonstrated that metformin might even reduce the risk of cardiac failure morbidity and mortality in diabetic patients (1,2,11,12). To find out the effects of adding metformin to patients on insulin therapy in the management of hyperglycemia in critically ill patients, 33 traumatized adult patients were randomly assigned to receive one of three protocols including intensive insulin monotherapy (A), metformin monotherapy (B), and intensive insulin therapy in combination with metformin (C) to maintain blood glucose level between 80-120 mg/dl. The results showed that metformin was cable of reducing insulin requirements in hyperglycemic patients independent of its plasma concentrations. It was concluded that metformin might be capable of reversing the insulin resistance without induction of lactic acidosis (13). On the other hand, it is possible that the use of metformin would be favorable in many with chronic renal failure based on the advantages of the reducing the metabolic syndrome and cardiovascular complications. Severe lactic acidosis in the absence of chronic kidney failure has raised the question that whether it needs to limit metformin use in these patients (1,2,11,12). Diabetic nephropathy is an important complication of diabetes mellitus and metformin is widely used for the treatment of type 2 diabetes (14). A study using metformin for diabetic rats for 17 weeks found that diabetes therapy with metformin restores podocyte loss. It was suggested that diabetes-induced podocyte loss could be suppressed by metformin through the repression of oxidative injury (15). According to previously published works and our results, metformin protects against tubular injury by modulation of oxidative stress on the tubules and restoring the biochemical alterations. Moreover, due to glycosuria in diabetes, there is tubular cell injury, which may protected by metformin. Metformin also able to protect podocytes in diabetic nephropathy (14-16). These findings may suggest the clinical use of metformin in the prevention of diabetic nephropathy. To better understand metformin renal protective effects, more preclinical and clinical studies are recommended.

## Author’s contribution

MRK is the single author of the manuscript.

## Conflict of interests

The author declared no competing interests.

## Ethical considerations

Ethical issues (including plagiarism, data fabrication, double publication) have been completely observed by the author.

## Funding/Support

None.
